# Effectiveness of estuarine adaptation strategies under future climate conditions

**DOI:** 10.1038/s41598-026-43040-7

**Published:** 2026-03-21

**Authors:** Johannes Pein, Joanna Staneva

**Affiliations:** Institute of Coastal Systems Analysis and Modelling, Helmholtz-Zentrum Hereon, Geesthacht, Germany

**Keywords:** Sea level rise, Climate change adaptation, Estuarine management, Eutrophication, Estuarine circulation, Numerical modelling, Climate sciences, Ecology, Ecology, Environmental sciences, Ocean sciences

## Abstract

Estuaries are among the most intensively used aquatic environments, where steep physical and biogeochemical gradients interact with dense human populations and valuable ecosystems. Intense human usage has historically led to ecological degradation and persistent use conflicts, in many temperate estuaries that host major inland ports such as the Elbe estuary in the North Sea. Climate change, with its associated effects on sea levels and global temperatures, represents a significant challenge for the functioning of these systems. The impact on estuarine hydrodynamics, sediment dynamics and water quality is, however, not yet fully understood. In this study, we utilise a coupled physical–biogeochemical model to assess the estuary’s response to an extreme climate scenario, focusing on the evaluation of potential adaptation options that could mitigate the impact of the climate scenario. By comparing changes in key physical and biogeochemical state variables across interventions, we assess whether river engineering measures can alleviate risks such as elevated storm-surge levels, enhanced upstream particulate transport, and climate-driven degradation of ecological and water-quality conditions. Our findings demonstrate that targeted adaptation can effectively mitigate several adverse consequences of sea level rise and warming, emphasising the necessity to integrate climate projections and adaptation pathways into future estuarine management strategies.

## Introduction

Estuaries have historically been coveted areas for settlement due to their high biological productivity and convenient accessibility^1^. As a vital connection between inland regions and the ocean, they serve as ideal transport routes. These advantages have led to estuaries being manipulated by humans for their own purposes since at least the Middle Ages to optimise navigability and other economic uses, therefore causing profound changes in the physical and ecological characteristics^2^. Today, these transformed landscapes fulfil both economic and ecological roles, yet balancing these interests remains a formidable challenge, often prioritising economic gains at the expense of ecological integrity^3^.

Recent decades have seen unparalleled climate and environmental shifts, leading to a broad consensus among scientists, policymakers, and society that current pathways are not viable^4,5^. Of particular concern is ongoing and projected sea level rise (SLR), which is expected to exceed 0.8 m by 2100 under high-end IPCC scenarios^6^. SLR raises the baseline water level on which storm surges are superimposed, increasing extreme water levels and reducing the capacity of existing flood defences to provide protection in the future^7^. This recognition underscores the need for estuaries—especially highly engineered ones in regions of pronounced sea level rise^8^—to explore effective adaptation options that mitigate potential future flooding hazards^9^.

**Fig. 1:**
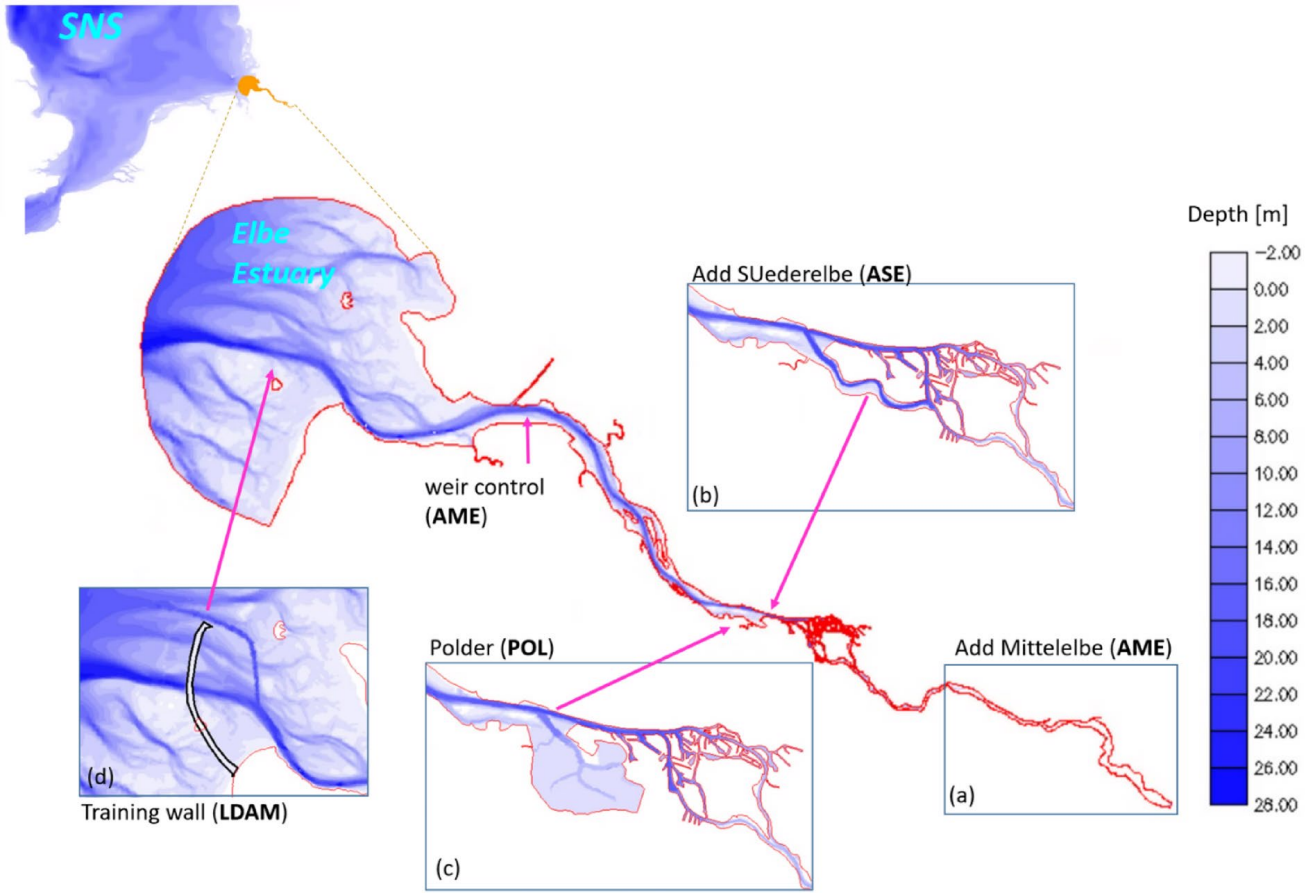
Model nesting system with topographies given for the Elbe estuary (center) and the intermediate model if the southern North Sea (“SNS”, top left) used to derive physical boundary conditions to the Elbe estuary. The boxes represent topographic features of the sustainable adaptation options (**a**) relocation of the tidal weir (“AME”), (**b**) opening of the historic channel branch Alte Suederelbe (“ASE”), (**c**) installation of a flood polder (“POL”), (**d**) construction of a sea wall (“LDAM”).

Beyond high water levels, SLR is widely known to alter salinity distribution and estuarine mixing^10^. A well-known potential hazard is the upstream propagation of the salt intrusion limit under rising sea level and reduced river discharge^11^. Increased salinity intrusion affects freshwater habitats, species distribution, and water usage for drinking and irrigation, making it a central variable that any long-term adaptation strategy must consider^12^. (Fig. 1).

Changes in water level and tidal propagation have equally strong implications for sediment dynamics and the morphodynamic evolution^13^. Theoretical relations between tidal prism and sediment budget suggest SLR-driven increases in cross-sectional area may promote sediment export^14^. However, in many modified estuaries, larger tidal prisms enhance tidal currents, bed shear stresses, and resuspension^10,15^, which—combined with the acceleration of the flood wave—can intensify tidal pumping and upstream sediment transport^16^, increasing turbidity and siltation in navigation channels^17^. A comparison across estuaries indicates that SLR can aggravate sediment accumulation in upper reaches^18^, illustrating the broader relevance of sediment-related hazards under climate change. In the Elbe estuary, where dredging is a permanent requirement to maintain navigability, understanding whether SLR amplifies these tendencies is a prerequisite for designing effective interventions.

Warming and hydrodynamic changes further influence biogeochemical functioning and oxygen dynamics. Higher temperatures accelerate heterotrophic respiration, remineralisation, and nitrification, increasing oxygen consumption rates^19^. Concurrently, increased turbidity from resuspension reduces light availability and could suppress primary production^20^, particularly in deepened freshwater reaches like the Port of Hamburg as reported from similar systems^21^. This combination points toward an increased risk of hypoxia during warm, low-flow periods—already observed in recent summers^22^—and highlights oxygen dynamics as a fourth critical hazard domain for estuarine adaptation planning.

While many estuaries of the southern North Sea are under the combined pressure of climate change and human activities, the Elbe estuary stands out due to its centuries-long history of human intervention and notably high eutrophication load. Historical disputes over river engineering privileges date back to the sixteenth century, and since the 1970s, regular channel deepening has accelerated physical transformations and sparked extensive scientific and societal debates^23^. As a result, the once largest inland delta in Europe, characterised by weak tides and limnic conditions, has transformed into an almost macrotidal waterway, increasingly influenced by marine climate and extremes. The upper end of the Elbe estuary, marked by a tidal weir, receives high levels of algae from the Middle Elbe, which is considered the federal waterway with the highest algae concentrations in Germany^24^.

From a socio-economic perspective, the Elbe Estuary presents complex ecological, economic and social challenges^25^, generating vigorous regional and international research interests^14,26^. There is an increasing consensus that river engineering practices, such as deepening, widening and straightening of the main channel between the Port of Hamburg and the lower estuary, have induced tidal pumping, high turbidity and siltation in the port area^23,27^. Furthermore, high organic inputs from upstream exacerbate issues like high turbidity and low oxygen levels, trends that are further fueled by prolonged drought since the 2010s and increasingly complex maintenance dredging^28^. Despite numerous initiatives and studies, achieving scientific and social consensus on sustainably managing the estuary to mitigate summer hypoxia, fish mortality, and the substantial economic and ecological costs of dredging remain elusive. This uncertainty parallels neighboring estuaries like the Ems Estuary or the Scheldt Estuary^26,29^, questioning whether there is a chance for the Elbe Estuary to remain a habitable place under the conditions of projected twenty-second century global warming and sea level rise.

Various scientific endeavors and social initiatives have proposed strategies to improve the ecological function of the Elbe estuary. These include creating expansive shallow water areas to improve oxygenation^26^, the opening of smaller inter-tidal areas along the estuary (so-called “Forum Tide-Elbe”, see https://www.forum-tideelbe.de/massnahmen/massnahmenkatalog) and the reduction of nutrient inputs into the Elbe River.

In our preceding study^30^, we systematically investigated how alternative estuarine geometries could mitigate present-day challenges in the Elbe estuary under historical forcing and contemporary physical and biogeochemical boundary conditions. That work applied a consistent modelling framework to assess several structural adaptation options—originally motivated by long-term estuary planning—designed to influence tidal propagation, storm surge attenuation, salinity intrusion, sediment dynamics, and estuarine biogeochemistry. The options addressed have been applied to other systems before, such as 1) dynamical weir control^31^, 2) reconnection of historic channel branch^32^, 3) adding a polder or retention basin to the tidal channel^33^, and 4) construction of a dam^34^. These studies in general indicate feasibility of the type of intervention, indicating that 1) active weir control in a deepened meso-tidal estuary reduces tidal amplitudes and dampens extreme water levels, thereby helping to moderate erosive hydrodynamics. The authors also reported, however, that long-term sustainability depends on balancing flood safety with the risk of increased sedimentation and ecological alteration caused by altered tidal propagation. 2) Intertidal areas reconnected to the main channel developed tidal channel networks that remained geomorphically simplified compared to the natural reference morphology. This limited their ability to fully recover the natural drainage and sediment distribution functions. The study indicates that historical diking imposes persistent constraints on long-term ecological and geomorphological sustainability, even after breaching. 3) Retention basins (polders) were found to significantly modify tidal dynamics by reducing tidal range and altering flow asymmetry. This has the potential to decrease sediment import and mitigate turbidity. The authors reported that, in the long term, such basins may enhance sustainability by weakening the hyperturbid state of tide-driven, industrialised estuaries, although they also risk disrupting natural sediment and habitat processes if not carefully designed. 4) A dam placed within the estuarine system strongly affected hydrodynamics, sediment transport and channel morphology, with these impacts being highly sensitive to the dam location and the timing of flows. The authors found that, while optimised operation could reduce sediment accumulation and stabilise morphology, poorly selected configurations might intensify siltation or disrupt ecological connectivity, thereby challenging long-term estuarine sustainability.

Acknowledging the potential feasibility of above river engineering schemes to modify estuarine dynamics our novel contribution was to compare them applying them in a single system^30^. Our hindcast simulations demonstrated that these structural measures induce substantial, yet highly non-linear, changes in hydrodynamics, salinity distribution, sediment transport, and biogeochemical functioning. They also revealed trade-offs rather than universally beneficial outcomes, with modifications improving certain sectors while exacerbating others. Importantly, variations in estuary shape strongly affected ensemble spread and internal variability, highlighting the need to understand these responses under future climate conditions.

**Fig. 2:**
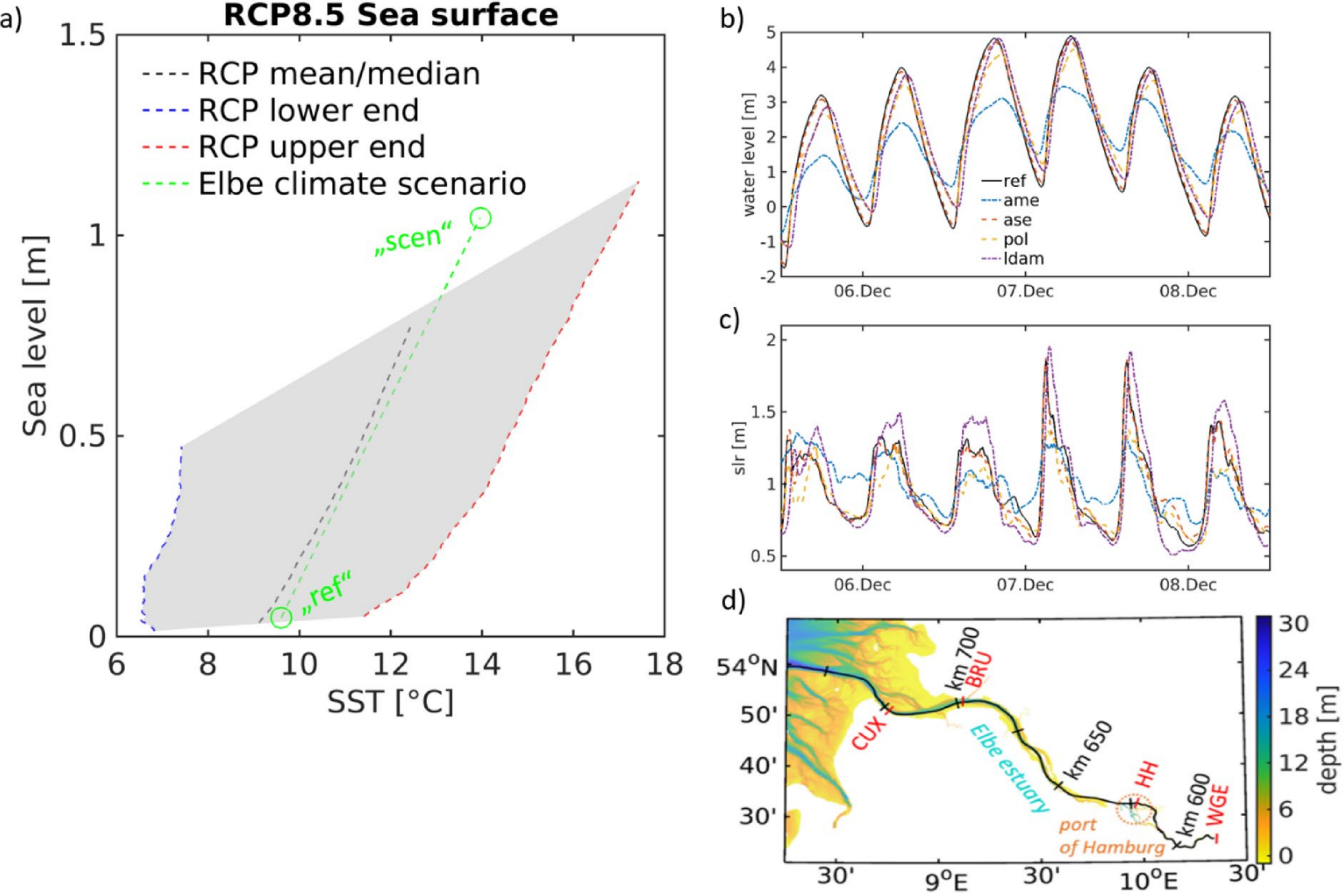
(**a**) mean sea level (MSL) over mean sea surface temperature (SST) in the RCP8.5 scenario with MSL trajectories at the Elbe estuary mouth from Palmer et al. (2018) and North Sea SST trajectories from the NOAA CMIP5 projection tool (https://psl.noaa.gov/ipcc/ocn/timeseries.html). Dashed blue, black and red lined represent lower, mid and upper end trajectories. Green circs mark the MSL and SST at the open ocean boundary in Elbe estuary hindcast run (“ref”) and climatic change scenario (“scen”), respectively, whereas the green dashed line is a linearly interpolated trajectory between the circs. Note that the SST data has been linearly extrapolated between 2090 and 2100 to be paired with MSL projection of Palmer et al. (2018). (**b**, **c**) Simulated water level oscillations in central port during storm Xaver (December 2013) are given (b) for reference run and adaptation scenarios. (c) as differences between climatic change scenario and simulation runs for the historic period in (b) representing the sea level rise signal. (d) gives the default topography of Elbe estuary along with relevant for analysis and interpretation locations, such as Cuxhaven (“CUX”), Brunsbüttel (“BRU”), Hamburg (“HH”) and weir Geesthacht (“WGE”). An orange circ marks the area of the port of Hamburg, the focal area to this study.

Building on the same modelling system and geometry variants, the present study shifts from a hindcast perspective to a scenario-based assessment, applying an extended RCP8.5 climate forcing to examine how these previously analysed adaptation options perform under sea level rise and global warming. The extended high-end climate change scenario is represented by increased mean sea level and global temperatures to be expected under an extreme, however possible, scenario after the year 2100 (Fig. 2a). Although studies on estuaries and coasts under climate change have tackled SLR before^35,36^, few have investigated cause-effect chains involving physics-biogeochemistry^37^, or even physics-biogeochemistry and human intervention in an overarching, cross-sectoral approach^38^. The aim of this study is to combine sea level rise and global warming in an extreme climate scenario and to calculate, compare and evaluate the changes in physical-biogeochemical impact chains through typical adaptation options tailored to the specific system against the backdrop of climate change in a region subject to ongoing observed and projected sea level rise and warming^8,22,36^. The overarching goal is to assess whether and how the proposed interventions could provide a effective solution in the event of an extreme climate, for example by reducing the height of peak storm surges and thus flood risk, mitigating the transport of particulates upstream that would necessitate additional maintenance work, and attenuating the negative consequences of higher water levels and temperatures on estuary ecology and water quality. This approach aims to achieve a new level of holistic scenario modelling by combining the individual considerations of the previous studies^31–34^, enabling an integral evaluation and discussion of adaptation pathways. A full description of the adaptation options is provided in our earlier work^30^, comprehensive methodological documentation and justification are therefore provided in the *Methods* section.

Following the overall aim, the specific scientific questions addressed in the following comprise: (1) How do the estuary’s physical and biogeochemical variables—including water levels, temperature, salinity, stratification, sediments, organic matter, plankton and oxygen—respond to the imposed climate change scenario? (2) How do alternative adaptation strategies modify climate-driven changes in storm surge height and tidal dynamics compared to the reference configuration? (3) How do these strategies affect broader physical responses to climate change, such as salinity intrusion, sediment transport, stratification and associated material fluxes? Finally, (4) how do the adaptation strategies influence the biological and water-quality consequences of climate change, particularly with regard to shifts in phytoplankton, grazers, particulate organic matter and bottom-oxygen concentrations in the critical upper estuary region? By aligning the analysis with these four domains, this study provides a holistic and sector-crossing assessment to determine whether effective adaptation pathways exist for the Elbe estuary under projected 22nd-century conditions.

## Results

In the following assessment, we analyse how the estuary responds to the imposed climate-change scenario and how a suite of adaptation options modifies this response. Alongside the reference geometry, we evaluate four proposed interventions—relocating the tidal weir (AME), reopening the historical *Alte Süderelbe* (ASE), constructing a large flood-storage polder (POL), and implementing a large diversion sea dike to deflect the incoming tidal wave (LDAM)—using the labels shown in the figures.

The response of storm-surge water levels to the different geometries is illustrated using the “Xaver” event causing the worst storm surges for decades in the North Sea on 5–6 December 2013, shown for the central port at station HH (Fig. 2d). In the historical simulations, peak water levels differ substantially among adaptation strategies (Fig. 2b). The reference and LDAM configurations follow similar trajectories with minor phase lags, while ASE lowers the peak of 4.89 m to 4.76 m. POL and AME produce the strongest reductions (4.52 m and 3.45 m, respectively), with AME also showing markedly elevated low-water levels, consistent with its ~ 70% reduction of flood volume in the lower estuary. Under future climate forcing, all adaptation strategies shift upward by ~ 1 m at surge peak, reflecting the imposed rise in mean sea level (Fig. 2c). However, their dynamic responses diverge. In the reference case, LDAM and ASE configurations, mid-flood water levels increase by nearly 2 m relative to the historical simulation, indicating substantially accelerated flood-wave propagation. By contrast, POL and AME show weaker mid-flood amplification (< 1.4 m), implying reduced sensitivity to enhanced marine forcing.

During the falling tide, however, differences between historic and future periods decrease to < 1 m across all configurations, indicating a faster ebb phase under climate forcing. Such accelerated ebbing is relevant for series of storm surges, where reduced time windows for hinterland drainage elevate residual-flood risks^9^. In this respect, POL is notable because it both reduces peak surge levels (Fig. 2b) and enhances ebb-phase water export relative to the reference case (Fig. 2c), increasing the system’s capacity to drain between closely spaced surge events. AME similarly lowers peak water levels and slows the rate of flood rise, although in contrast to POL it produces a comparatively slower post-peak drainage, reflecting the intentionally restricted flood volume^30^.

Overall, the adaptation strategies (Fig. 1a-d) demonstrate lower peak surge levels under both historic and emulated climate change conditions, making them preferable to the business-as-usual strategy represented by the reference geometry. POL offers the most balanced benefit reducing peak surge heights (Fig. 2b), delaying the flood rise, and enhancing ebb export (Fig. 2c). AME provides similar peak-level reductions but drains more slowly after surge peak. Thus, Fig. 2 demonstrates how the four adaptation options modify climate-driven changes in storm-surge height and tidal dynamics (Research questions 1 & 2).

**Fig. 3:**
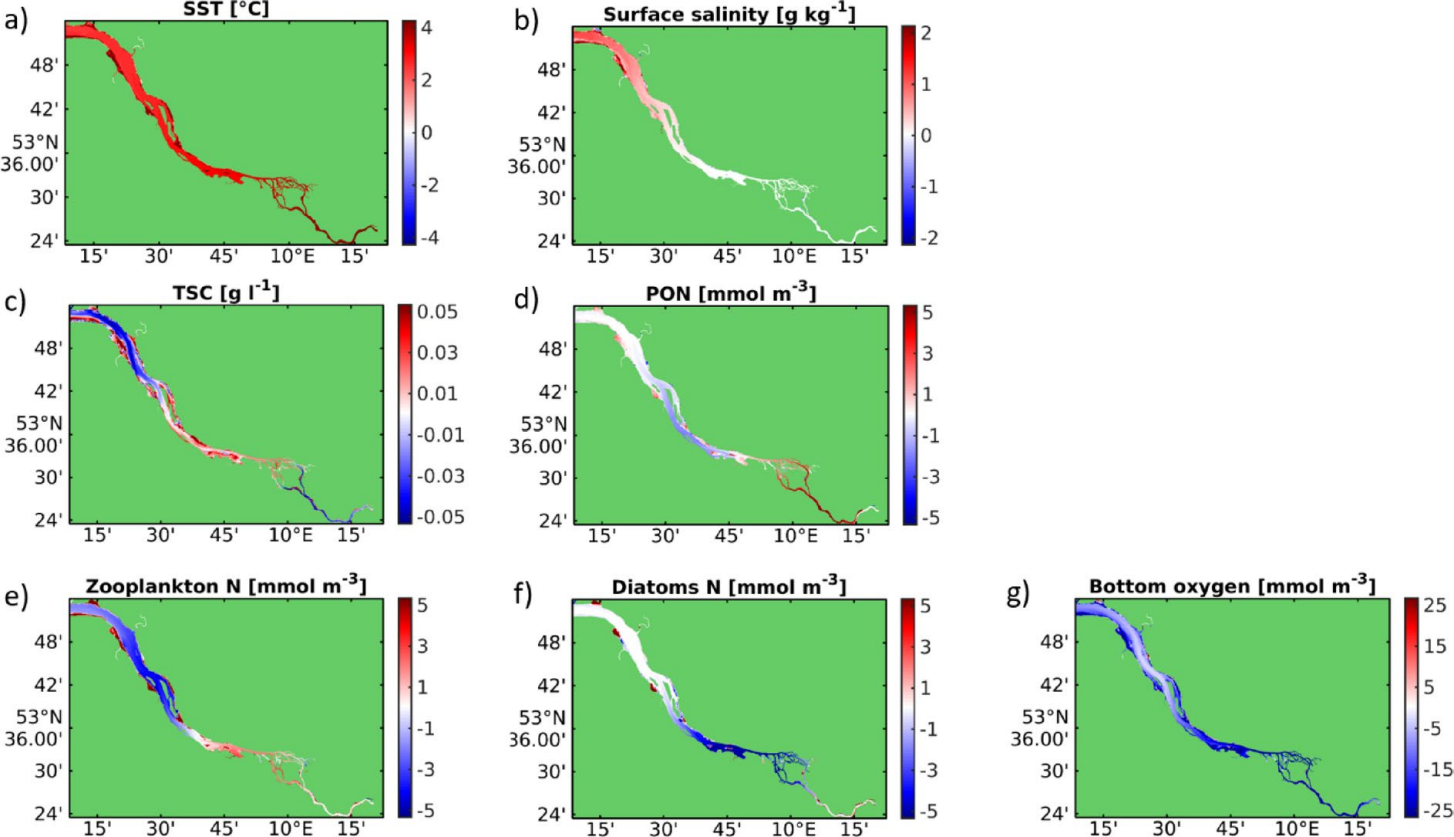
Average differences of (**a**) surface temperature, (**b**) surface salinity, **c** suspended sediment, (**d**) phytodetritus/particulate organic nitrogen (PON), (**e**) zooplankton biomass nitrogen, (**f**) diatom biomass nitrogen, (**g**) bottom oxygen concentration between climate scenario and reference run during May to September illustrate the essential effects of global warming and sea level rise in the estuary.

The imposed warming and sea level rise cause systematic shifts in estuarine hydrography (Fig. 3). Estuarine water surface temperatures increase by ~ 3.2 °C on average in summer (Fig. 3a), while higher water levels and enhanced horizontal velocities (not shown) intensify salinity intrusion (Fig. 3b). Increased salinity strengthens vertical density gradients locally, suppressing turbulence and vertical mixing while reinforcing the estuarine circulation. These changes set conditions for downstream reductions in surface sediment concentrations due to damped turbulence (Fig. 3c), contrasted by enhanced resuspension in the deepened freshwater reach and lower port. Enhanced flood-wave celerity (Fig. 2c) strengthens landward sediment transport, increasing tidal pumping and shifting mineral and organic particulates upstream (Figs. 3c, d). While inorganic sediment concentrations increase in the deepened freshwater reach and up to the lower port (Fig. 3c), the lighter particulate organic matter is transported even farther upstream, accumulating in the shallow upper estuary (Fig. 3d). Zooplankton and phytoplankton respond consistently with these physical changes (Fig. 3e, f). Under future climate conditions, phytoplankton biomass shifts landward, closely mirroring the upstream displacement of both mineral and organic suspended matter (Figs. 3f, c, d). This redistribution likely reflects increased residence times, enhanced stratification, and the stronger tidal pumping associated with higher flood-wave celerity (Fig. 2c). The concurrent upstream shift of grazers (Fig. 3e) further reinforces this pattern through trophic coupling. These patterns confirm that climate-driven changes in tidal prism, straining, and wave celerity substantially reorganize sediment and organic-matter dynamics (Research Question 3). The upstream accumulation of organic material sets up conditions for increased control oxygen depletion at the transition from the deepened freshwater reach to the shallow upper estuary.

**Fig. 4:**
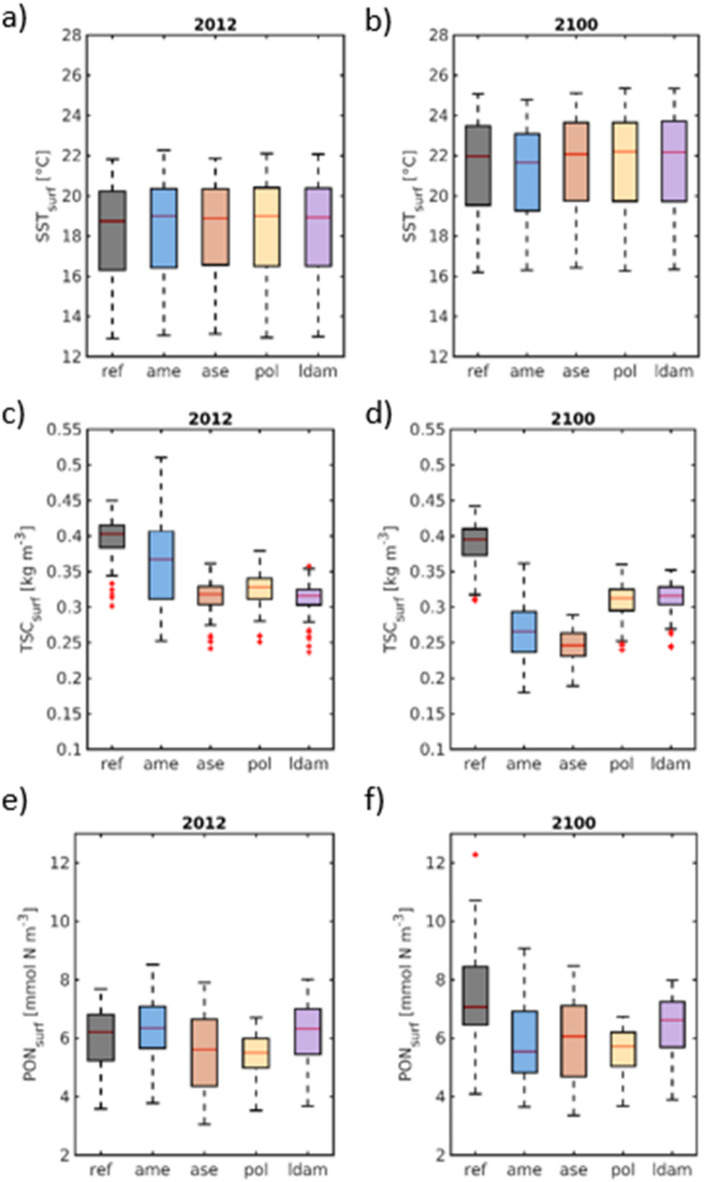
Boxplots of time- and space-averaged main channel (**a**, **b**) surface temperature, (**c**, **d**) sediment concentration and (**e**, **f**) phytodetritus/particulate organic nitrogen concentration during (a, c, e) May to September 2012 and during (b, d, f) the same period in a climatic change scenario representing projected changes to sea level rise and sea temperature after the end of the century (“2100”). The data have been sampled between km 600 and km 650 along the black transect line depicted in Fig. 2d. Note that the individual boxplots’ central mark indicates the median, and the bottom and top edges of the box indicate the 25th and 75th percentiles, respectively. The most extreme data points are represented by the whiskers.

This region (km 600–650)—being most affected by present-day low-oxygen conditions^22^—is subject to profound changes under the climate scenario and will be focused on in the following comparison and assessment of adaptation options. In this reach, surface temperatures increase by ~ 3 °C across all adaptation strategies, with only minor ensemble spread (4a, b), intensifying metabolic rates and oxygen demand. The response of suspended particulate matter is more complex. In the reference case, surface inorganic sediment concentrations in the oxygen-minimum region remain close to historic values (Figs. 4c, d). This stability results from two compensating effects: although resuspension intensifies in the deepened freshwater reach (Fig. 3c), sediment concentrations decrease in the upper port and shallow upper estuary (Figs. 2d and 3c), reducing the net supply of suspended sediments into the oxygen-minimum zone. Because elevated suspended sediments are a known driver of bottom-oxygen depletion via enhanced turbidity^30^, reduced primary production and stimulated remineralisation, this spatial rearrangement partially offsets the climate-driven risk of further oxygen decline.

The adaptation strategies diverge from the reference case in several notable ways. The AME, ASE and POL configurations demonstrate a similar trend of decreasing median sediment concentrations, with the strongest reductions being produced by ASE and AME—from 0.34 to 0.25 kg m⁻^3^ and from 0.35 to 0.30 kg m⁻^3^, respectively. In contrast, the LDAM configuration slightly increases inorganic sediment concentrations (from 0.32 to 0.33 kg m⁻^3^), which is consistent with the enhanced upstream pumping observed under this geometry. Particulate organic nitrogen (PON) increases markedly across the region under future conditions (Fig. 4e, f), reflecting the upstream trapping of organic material (Fig. 3d). All four adaptation strategies mitigate this accumulation to varying degrees, likely because their geometries reduce the amplification of marine forcing—and thus tidal pumping—associated with higher sea level. This effect is either direct (AME, LDAM) through modifications of the incoming tidal wave, or indirect (ASE, POL) through a reduced convergence at the entrance to the port (Fig. 1; orange circle in Fig. 2d). Here, in the tidal freshwater reach upstream of km 645, the tidal prism amounts to 3.24 × 10⁶ m3 in the reference case and increases by 11% and 28% under the ASE and POL adaptation options, respectively, reflecting the associated changes in channel cross-section and storage volume.

**Fig. 5:**
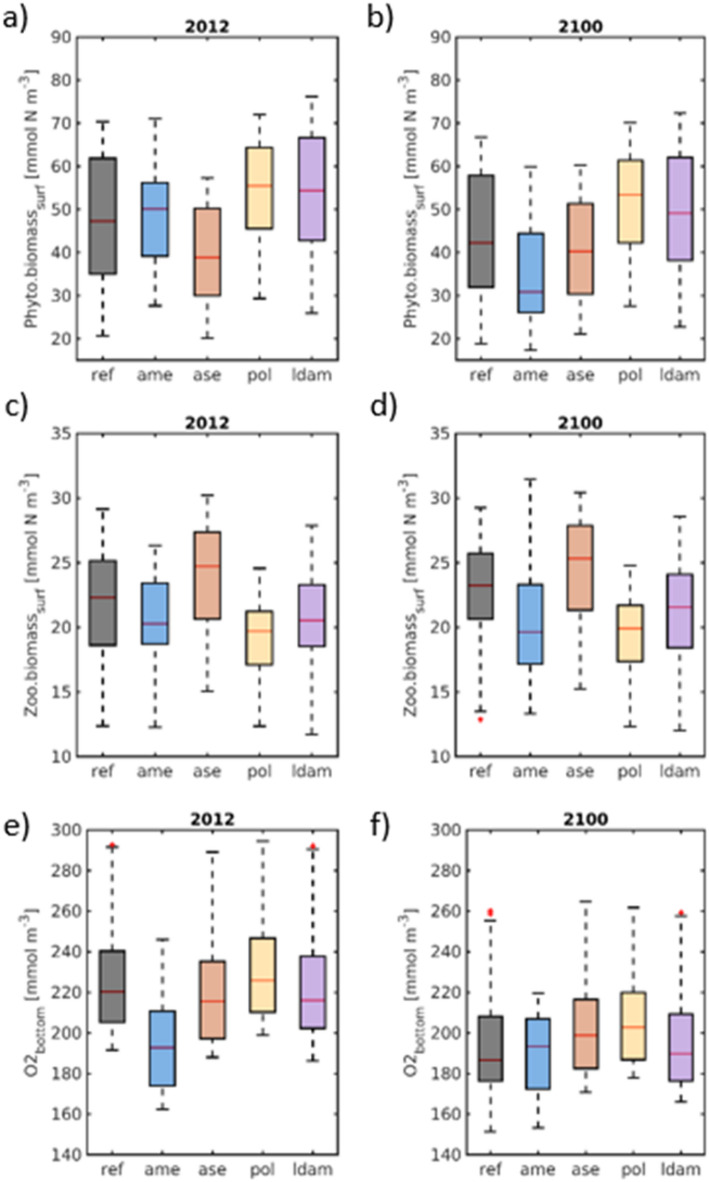
Boxplots of along-channel (**a**, **b**) surface phytoplankton biomass nitrogen, (**c**, **d**) surface zooplankton biomass nitrogen and (**e**, **f**) bottom oxygen concentration during (a, c, e) May to September 2012 and (b, d, f) during the same period in a climatic change scenario representing projected changes to sea level rise and sea temperature after the end of the century (“2100”).

Phytoplankton biomass shows diverse responses. The reference geometry and LDAM exhibit moderate decreases (Fig. 5a, b), whereas AME shows a pronounced decline (~ 40% reduction of median values), consistent with reduced light availability and altered residence times. ASE, by contrast, produces a slight increase in phytoplankton biomass, likely linked to reduced sediment concentrations and improved light conditions (Figs. 4c, d). Grazer biomass follows a similar pattern, with ASE again being the only configuration that mildly increases grazer concentrations under future conditions (Fig. 5c, d). Bottom oxygen concentrations decrease across all adaptation options under future climate forcing (Fig. 5e, f), mainly due to warming-enhanced respiration and reduced diatom production (Fig. 3f). The reference configuration exhibits the strongest decline (from 220 to 185 mmol m⁻^3^; ~ 6 mg l⁻^1^), with maximum reductions exceeding 44 mmol m⁻^3^ near km 607. Accumulated PON (Figs. 3d, 4e, f) and weakened turbulent mixing amplify this decrease. AME and ASE partially compensate for these stressors by reducing sediment resuspension (Fig. 4c, d), lowering PON accumulation (Fig. 4e, f), and moderating stratification. POL maintains the highest bottom-oxygen concentrations among all adaptation strategies in the future-climate ensemble, slightly outperforming ASE. These results show that climate-driven oxygen depletion is substantial but can be mitigated through targeted adaptation strategies—answering the fourth research question.

## Discussion

A high-end climate-change scenario combining elevated mean sea level and increased global water temperatures produced a consistent intensification of estuarine dynamics, including higher tidal water levels and amplified storm-surge peaks in the upper estuary. The adaptation strategies mitigated both the historical storm “Xaver” and its climate-amplified counterpart, with the tidal-weir and flood-polder configurations providing the strongest reductions in peak surge levels or the most efficient post-surge drainage, respectively^9^. These results are consistent with previous studies showing that measures such as controlled flood-storage areas, targeted modifications of tidal prism, and selective hydraulic constrictions can reduce estuarine vulnerability to marine hazards^31–34^. The altered post-surge drainage response under the climate scenario shows that the performance of flood-defence adaptation measures is climate-state dependent, highlighting the need to evaluate such measures under projected future mean sea levels. On seasonal time scales, the climate scenario produced stronger salinity intrusion and increased particulate transport into the deepened freshwater reach and port, while phytoplankton declined and zooplankton increased. Notably, all proposed adaptation strategies exhibited increased effectiveness in mitigating the upstream accumulation of particulate organic matter under the climate scenario. The system-wide deterioration of bottom oxygen levels reflects a characteristic warming response of coastal ecosystems^37^, with the strongest impacts located between km 600 and km 650, a reach known to be sensitive to stratification, organic loading, and human modification^5,14,17,26^.

The reference configuration experienced the most severe losses in phytoplankton biomass and bottom-oxygen concentrations, whereas the polder option and the reconnection of a historic channel branch provided the most resilient outcomes, with the latter proving effective in mitigating bottom-oxygen decline only under the climate change scenario. All proposed adaptation configurations appear to mitigate the climate-induced amplification of marine forcing—either by directly modifying tidal propagation or by reducing channel convergence with associated increases in tidal prism—and thereby limit organic-matter trapping, stratification, and bottom-oxygen decline. Their performance is consistent with theoretical work suggesting that restoring entropy production via enhanced mixing can improve resilience under climate warming^39^. Notably, the reconnection option was the only configuration that increased grazer biomass under climate forcing, showing that restoring lateral connectivity and hydraulic diversity can strengthen trophic resilience even when primary production declines.

This study highlights that assessments of estuarine adaptation options should not be limited to contemporary boundary conditions, extending earlier insights on the importance of forward-looking management^26,31,34^. Evaluated under projected future marine forcing—including higher water temperature and sea level^36,37^—some adaptation options that appear only moderately effective today become viable pathways under future conditions. This result is highly relevant for estuarine planning, where decision-making horizons extend across decades and climate-driven changes will progressively reshape tidal dynamics, sediment pathways, and biogeochemical functioning. While the ad hoc scenario design omits transient climatic and management trajectories, incorporating such complexity would introduce numerous possible physical–biogeochemical bifurcations. The simplified approach used here therefore offers clear, interpretable insights into how different adaptation pathways diverge under climate change and provides an essential scientific basis for long-term estuarine management and societal decision-making.

## Conclusions

Our findings emphasise the vital impact of rising sea levels on estuarine ecosystems, particularly in the context of climate change. Our simplified climate change scenario, which involved enhanced mean sea levels and global water temperatures, led to intensified tidal heights and storm surge peaks in the upper estuary’s port region. Moving forward, it is crucial to consider these adaptation strategies not only against current conditions but also in light of projected future climate scenarios. Effective estuarine management requires ongoing monitoring and mitigation efforts to safeguard ecosystem health, particularly in transitional zones vulnerable to salinity intrusion and sedimentation. Our study also emphasises the importance of robust numerical modelling to inform decision-making processes, illustrating potential divergent trajectories under different adaptation pathways. As communities plan for climate adaptation, understanding these dynamics will be crucial for devising effective strategies that enhance estuarine resilience and mitigate the impacts of sea level rise on coastal environments. The limitations to this study arise from the relatively short simulation time of up to one year^30^ in comparison with climatic time scales and in particular the absence of transient morphodynamics simulations that could however only be tackled in connection with transient modelling of socio-economic drivers and corresponding maintenance cycles in a managed system.

## Methods

### Epistemic approach based on adaptation strategies

This study takes a scenario-based approach, which considers and examines the estuarine response under contemporary conditions in comparison with future, projected forcing conditions for a range of different geometries and technical boundary conditions. We use the reference topography as the business-as-usual strategy (Fig. 1). It is based on the history of use and development of the Elbe estuary, as described in the Introduction, which it shares with other industrialized estuaries. We consider the adaptation strategies methodically as what-if scenarios, i.e., the change is introduced and considered ad hoc, not as a transient scenario. The four adaptation options considered are based on literature research on this and similar systems, as well as exchanges with stakeholders during project work and workshops on the topic of climate adaptation^30^. The adaptation options include the following.

- Relocating the tidal weir (AME, Fig. 1a): This option reconnects the Middle Elbe, upstream of the current tidal barrier, with the main estuary, while relocating the tidal weir downstream into the salinity-front region. The passive structure is replaced by an actively operated sluice that is partially closed (70%) during the flood and fully open during the ebb, in line with the Ems estuary scheme. The new weir at Elbe km 692 consists of 12 gates with a total length of 1.5 km, while the inflow of freshwater upstream remains unchanged.

- Reopening the historical Alte Süderelbe (ASE, Fig. 1b): This option restores and extends the former tidal branch to create a 13.3 km long, 300–400 m wide, 18 m deep channel that links the deepened freshwater reach with the shallow upper estuary. The channel bypasses the port area and provides an additional tidal pathway between the western main channel and the southern port channel.
- Constructing a flood-storage polder (POL, Fig. 1c): Here, the estuary is expanded by a large intertidal polder located downstream of Hamburg, near the historic Alte Süderelbe inlet. Following^26^, the polder adds approximately 4,800 hectares of tidal flats and channels with an average depth of around 1.5 m, converting part of the marshland into an intertidal zone.
- Building a sea dike (LDAM, Fig. 1d): This option involves constructing a large sea dike in the outer estuary, comparable to the dam existing the Ems estuary^17^, to deflect incoming Kelvin waves and storm surges. As this closes the existing ebb-delta navigation channel, a new dredged channel is required to connect the estuarine main channel with the northern tidal channel, in order to maintain navigation and river outflow.

To evaluate the feasibility of the four adaptation options—reopening the historical Alte Süderelbe (ASE), relocating the tidal weir (AME), constructing a large flood-storage polder (POL), and implementing a large diversion sea dike that deflects the incoming tidal wave (LDAM)—we designed a what-if scenario modeling framework in which each adaptation strategy was implemented as a distinct geometric modification of the reference estuary configuration. For all configurations, we defined consistent model inputs, including bathymetry, boundary conditions, hydrodynamic and biogeochemical forcing, and applied a uniform climate-change perturbation consisting of prescribed sea level rise, atmospheric warming, and enhanced marine boundary conditions. To address research question (1), each simulation was first run under both historic and climate-change forcing to quantify the estuary-wide physical and biogeochemical response attributable solely to climate change. The climate scenario was implemented by adding 1 m of mean sea level at the open boundary of the forcing model^16^, while warming was implemented by adding 4°C to the water temperature at all open boundaries and for the water surface forcing. To address question (2), the same climate forcing was applied to all adaptation geometries, allowing direct comparison of how storm-surge elevation and tidal dynamics differ from the reference case. Question (3) was approached by analysing strategy-specific changes in salinity intrusion, sediment dynamics, and material fluxes derived from the model output fields. Finally, for research question (4), we quantified the ecological and water-quality consequences of each adaptation option—focusing on phytoplankton, grazers, particulate organic matter, and bottom-oxygen concentrations—using the same evaluation metrics applied to the reference geometry. By explicitly aligning the numerical setup and diagnostic procedures with these four analytical domains, the study provides transparency, reproducibility and a consolidated basis for assessing whether effective adaptation pathways exist for the Elbe estuary under projected 22nd-century climate conditions.

### Numerical framework

The semi-implicit cross-scale hydroscience integrated system model^40^ has been employed by researchers to successfully model both idealised and realistic estuarine domains, addressing research questions in hydrodynamics. Further SCHISM studies addressed sediment dynamics and ecology^41–44^. The model predicts water level, horizontal currents, vertical exchange and tracer transport^40^. In the vertical direction, the model domain is resolved using terrain-following coordinates, while the turbulence closure is represented by the k-ε model of the GOTM turbulence model^45^. In the coupled configuration used in this study, the SCHISM predicts the transport and diffusion of ecosystem state variables and provides salinity, water temperature and bottom stresses to the ecosystem module.

Simulation of tidal weir control in SCHISM has been implemented by the Californian Department of Water Resources (see http://ccrm.vims.edu/yinglong/wiki_files/structs_main.pdf). For purpose of simulating a tidal weir in this exercise, we assume a simple rectangular sluice gate with flow transfer function

1 $$Q_{s} = {\mathrm{sgn}} (z_{u} - z_{d} )C_{op} C_{f} A\sqrt {2g|\Delta z|}$$

where $sgn ({z}_{u}-{z}_{d})$ is the sign of the water level difference between upstream and downstream of the weir, ${C}_{op}$ is a directionally varying operation coefficient, ${C}_{f}$ is a flow/gate coefficient, *A* is the area of the gate opening,* g* is gravity constant and $|\Delta z|$ is the water level difference upstream and downstream of the weir. Here, partial closure of the weir gates on flood is achieved by stipulating a directional operation coefficient of ${C}_{op}$= 0.3 on flood and ${C}_{op}$ = 1.0 on ebb.

Furthermore, the SCHISM framework incorporates a sediment and morphodynamic module, in addition to the hydrodynamic core. The sediment model, designated SED3D, has been derived from the Community Sediment Transport Model^46,47^. For the initialisation and parameterisation of the coupled hydrodynamic and sediment simulations (SCHISM-SED3D), observations of bottom sediment fractions provided by federal authorities via the portal “kuestendaten.de” are utilised.

The biogeochemical model is derived from the ECOSystem MOdel (ECOSMO^48,49^). In the coupled physical-biogeochemical model, the local concentration of an ecological tracer is given by the following equation:

2 $${C}_{t}+\left(\mathbf{v}\nabla \right)C+\left({w}_{d}\right){C}_{z}=\left({A}_{v}{C}_{z}\right)+{R}_{C},$$

where *C* is an ecological state variable, *v* is the 3D velocity field, *w*_*d*_ is a constant settling velocity and* A*_*ν*_ is the turbulent diffusion coefficient. The pelagic prognostic state variables C include four nutrients, three functional groups of primary producers, herbivorous and omnivorous zooplankton, detritus, opal and dissolved organic matter. The term *R*_*C*_ in Eq. 1 represents the biogeochemical sources and sinks that modify the respective state variable concentration C. The following description of the kinetic reactions in the biogeochemical model focuses on the most relevant heterotrophic turnover processes in estuaries (for details see^49,50^).

**Fig. 6:**
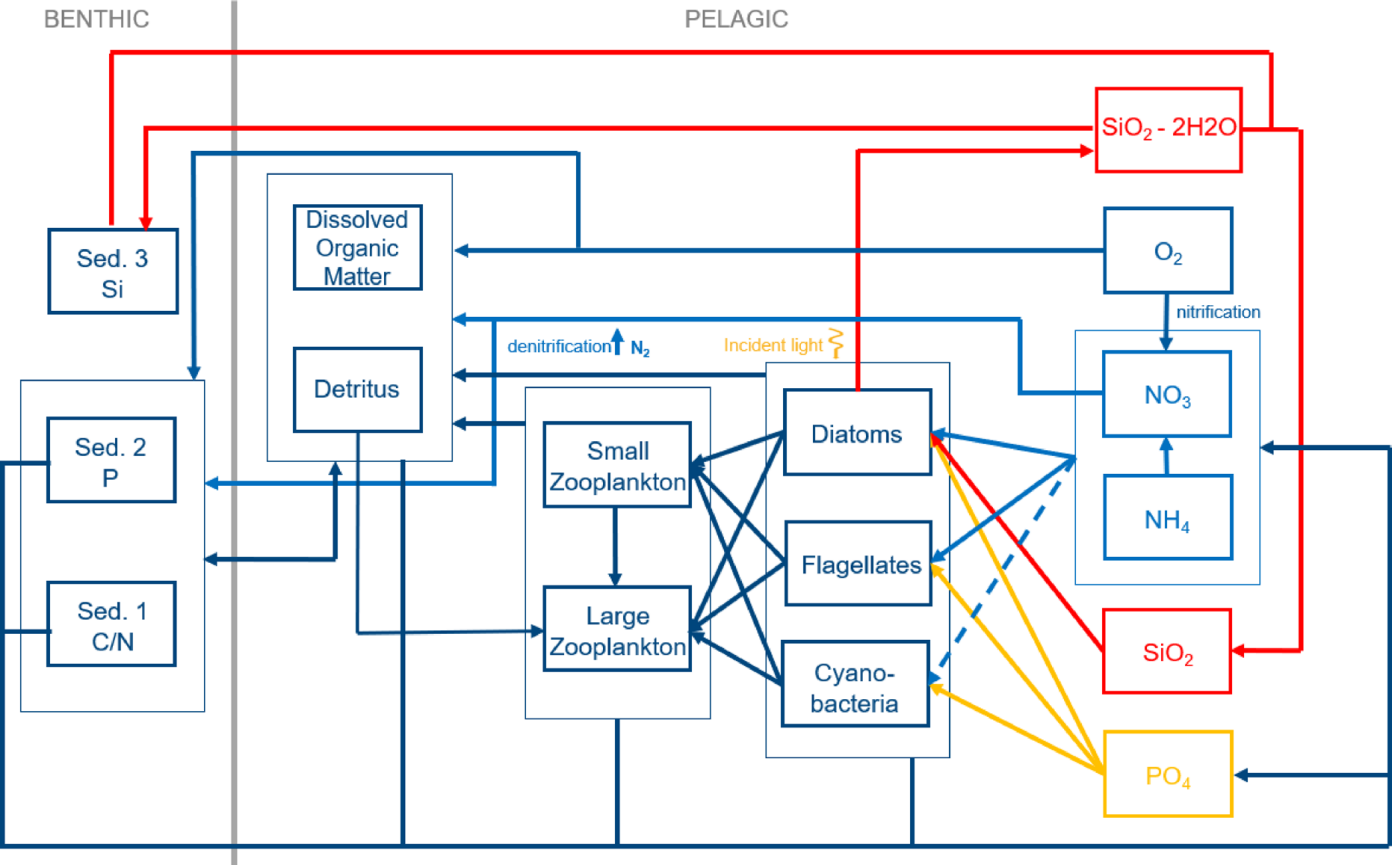
Links between the variables of the ecological module containing (columns from right to left): Nutrients and oxygen, primary producers, secondary producers, organic matter and sedimentary nutrient storage species (adapted from^44^).

Assuming Redfield ratio of nutrients^51^, ECOSMO implements limitation of primary production by the availability of nutrients and light^52^. Phytoplankton biomass decline occurs as a result of respiration and grazing. The latter is a function of the local concentration of zooplankton and the feeding preferences of zooplankton groups. The availability of light is dependent upon a background extinction coefficient and shading by particulates, including phytoplankton, phytodetritus and mineral sediments. Phytodetritus is subject to messy feeding by grazers and acts as a source of ammonium via remineralisation. The degree of nutrient limitation varies according to the functional group of primary producers. Diatoms require nitrogen, phosphorus and silicate, whereas flagellates are controlled by nitrogen and phosphorus availability alone. Cyanobacteria are nitrogen fixators and are limited by phosphorus availability only. In the configuration employed here, the simulation of inorganic sediment dynamics in SCHISM^42,47^ has been enabled as described in^53^. The links between the ecological state variables are shown by Fig. 6.

For the physical and economical assessment, simulations rely on the SCHISM-SED3D code available with the default version at the GitHub repository (https://github.com/schism-dev/schism). For the coupled physical-biogeochemical simulations for the ecosystem assessment we use the SCHISM(-SED3D)-FABM-ECOSMO framework developed and presented in^22,30,44^ using physical parametrisations from a recent work on coupled physical-biogeochemical processes in the Elbe estuary^22^. The computational mesh of the reference case (Fig. 1) consists of 78,370 mesh elements and 41,912 mesh nodes.

Forcing data consist of historical hydrodynamic, atmospheric and hydrologic reanalysis and observations. The oceanic forcing has been generated using a simulation of a larger model domain covering the southern North Sea^16^, following the nesting approach used in^30^. Namely, the nesting between the intermediate model and the estuarine model is performed as a one-way nest with a time resolution of 1 h. The atmospheric forcing and river discharge remain the same in both forcing (parent) and forced (child) model simulations. Elbe river discharge data and nutrient loads stem from environmental administration and have been downloaded from the repositories at https://www.kuestendaten.de/DE/Services/Messreihen_Dateien_Download/Download_Zeitreihen_node.html and https://www.elbe-datenportal.de/FisFggElbe/content/auswertung/MessstellenDetail_erstStart. Nutrient data have been complemented by observations from HEREON research center, Geesthacht (see details in^44^). The atmospheric forcing has been provided by the German Weather Service (DWD, https://www.dwd.de/DE/Home/home_node.html) and is based on the COSMO EU model^54^. Same forcing and model parametrisations have been used in^22^. The changed workflow and updated parametrisations consolidated the consistency and robustness of the modelling approach as demonstrated by validation of tidal parameters for reference simulation (Table 1) following a validation strategy proposed by^55^. Salinity, temperature and biogeochemical parameters have been extensively validated in previous studies^22,44^.

**Table 1:** Root-mean-square error for tidal parameters of water level in the Elbe estuary (following^26^), referring to root-mean square error (RMSE) of tidal range (TR), high water (HW), low water (LW) and mean water level (MW).

Location	RMSE of TR	RMSE of HW	RMSE of LW	RMSE of MW
Cuxhaven	13.4 cm	11.2 cm	13.5 cm	10.0 cm
St. Pauli	18.4 cm	14.8 cm	15.2 cm	10.6 cm
Mean of gauges	21.5 cm	14.7 cm	18.4 cm	11.9 cm

## Data Availability

The datasets used and/or analysed during the current study available from the corresponding author on reasonable request. The reference and scenario topographies, as well as the historic and climate boundary forcing used are available at the following repository [10.5281/zenodo.17786062] (https:/doi.org/10.5281/zenodo.17786062).
